# 5G: The Convergence of Wireless Communications

**DOI:** 10.1007/s11277-015-2467-2

**Published:** 2015-03-13

**Authors:** Raúl Chávez-Santiago, Michał Szydełko, Adrian Kliks, Fotis Foukalas, Yoram Haddad, Keith E. Nolan, Mark Y. Kelly, Moshe T. Masonta, Ilangko Balasingham

**Affiliations:** 1 Norwegian University of Science and Technology, Trondheim, Norway; 2 Intervention Center, Oslo University Hospital, Oslo, Norway; 3 Institute of Clinical Medicine, University of Oslo, Oslo, Norway; 4 Huawei R&D Sweden, Stockholm, Sweden; 5 Poznan University of Technology, Poznan, Poland; 6 Athena Research and Innovation Centre, Industrial Systems Institute, Patras, Greece; 7 Jerusalem College of Technology, Jerusalem, Israel; 8 Intel Labs Europe, Intel, Leixlip, Ireland; 9 Department of Electrical Engineering, Tshwane University of Technology, Pretoria, South Africa; 10 Meraka Institute, Council for Scientific and Industrial Research (CSIR), Pretoria, South Africa

**Keywords:** 5G, Radio spectrum, Traffic offloading, Small-cells, Software defined radio, Software defined networking

## Abstract

As the rollout of 4G mobile communication networks takes place, representatives of industry and academia have started to look into the technological developments toward the next generation (5G). Several research projects involving key international mobile network operators, infrastructure manufacturers, and academic institutions, have been launched recently to set the technological foundations of 5G. However, the architecture of future 5G systems, their performance, and mobile services to be provided have not been clearly defined. In this paper, we put forth the vision for 5G as the convergence of evolved versions of current cellular networks with other complementary radio access technologies. Therefore, 5G may not be a single radio access interface but rather a “network of networks”. Evidently, the seamless integration of a variety of air interfaces, protocols, and frequency bands, requires paradigm shifts in the way networks cooperate and complement each other to deliver data rates of several Gigabits per second with end-to-end latency of a few milliseconds. We provide an overview of the key radio technologies that will play a key role in the realization of this vision for the next generation of mobile communication networks. We also introduce some of the research challenges that need to be addressed.

## Introduction

The ongoing deployment of the fourth generation (4G) of wireless mobile systems has prompted some telecommunication companies to consider further development towards fifth generation (5G) technologies and services. Since the appearance of the first generation (1G) system in 1981, new generations have emerged approximately every 10 years. Wireless communication generations typically refer to non-backwards-compatible standards following requirements specified by the International Telecommunication Union-Radiocommunication Sector (ITU-R). Examples of these specifications are International Mobile Telecommunications 2000 (IMT-2000) for 3G and IMT-Advanced for 4G.

Other standardization bodies like IEEE also develop wireless communication technologies, often for higher data rates albeit shorter transmission ranges in most cases. With these additional standards, it is common that predecessor systems occur on the market a few years before new cellular mobile generations. For instance, commercial mobile Worldwide Interoperability for Microwave Access (WiMAX) networks deployed since 2006 are considered predecessors to 4G. Later, first-release Long-Term Evolution (LTE) systems were marketed as 4G in Scandinavia and the United States in 2009 and 2010, respectively. However, these networks were not strictly compliant with the original IMT-Advanced technical requirements. In contrast, the latest 4G LTE-Advanced (LTE-A) systems will fully comply with the ITU-R specification and support peak downlink data rates of 100 Mbps and 1 Gbps for vehicular and pedestrian mobility, respectively. Future 5G systems are expected to provide significant gains over 4G like higher data rates, much better levels of connectivity, and improved coverage. An overview of the technical solutions considered for the “beyond-4G” cellular system is provided in, e.g., [3]. Various aspects of the next generation networks have been addressed in recent papers, e.g., [1, 14, 40, 43, 58]. Finally, comprehensive vision of the 5G networks and even beyond is presented in [60].

Nevertheless, there is still some debate on the technical characteristics that 5G must possess and the services it will provide. 5G is not a term officially used in any particular specification or in any official document made public by ITU-R or any standardization body. Moreover, some industry representatives have expressed skepticism towards 5G. Others, however, envisage the rollout of 5G for the early 2020s [52]. Enabling technologies for the next generation have started to be researched by consortia comprising key international mobile operators, network infrastructure manufacturers, and academic institutions. For instance, in October 2012, the University of Surrey in the United Kingdom secured £35 million in funding for the 5G Innovation Centre, 5GIC (http://www.surrey.ac.uk/ccsr/business/5GIC/), which offers testing facilities to mobile operators developing more energy and spectrum-efficient technologies for data rates beyond 4G capabilities.

In November 2012, the EU Project “Mobile and wireless communication Enablers for the Twenty-twenty Information Society,” METIS 2020 (please see Table 1), started activities toward an international definition of 5G. In February 2013, the European Commission announced €50 million for research to deliver 5G mobile technology by 2020. Some recent EU-funded research projects that address the architecture and functionality for networks beyond 4G are listed in Table 1.

**Table 1:** Examples of EU Projects researching technology beyond 4G

Project name	Research area	Website
METIS 2020	Laying the foundation for the future global mobile and wireless communication systems	www.metis2020.com
5GNOW	Development of new PHY and MAC layer concepts better suited for heterogeneous transmissions in 5G	www.5gnow.eu
SOLDER	Design and development of new spectrum overlay technology for efficient aggregation of heterogeneous bands (HetBands)	www.ict-solder.eu
iJOIN	Joint design/optimization of access network and backhaul, integrating small cells, heterogeneous backhaul, and centralized processing	www.ict-ijoin.eu
TROPIC	Distributed computing, storage, and radio resource allocation over cooperative femtocells	www.ict-tropic.eu
MCN	Cloud computing for future mobile network deployment and operation	www.mobile-cloud-networking.eu/site
COMBO	New integrated approaches for fixed/mobile converged broadband access/aggregation networks	www.ict-combo.eu
MOTO	Mobile Internet with terminal-to-terminal offloading technologies	www.fp7-moto.eu
PHYLAWS	Privacy concepts for wireless communications exploiting propagation properties	www.phylaws-ict.org

On the regulatory side, the ITU-R Working Party 5D (WP 5D) started two study items in February 2013, aimed at obtaining a better understanding of future technical aspects for mobile communications towards the definition of the next generation: (1) a study on IMT vision for 2020 and beyond, and (2) a study on future technology trends for terrestrial IMT systems. In light of these developments, the European COST Action^1^ IC0905 “Techno-Economic Regulatory framework for Radio spectrum Access for cognitive radio/software defined radio,” TERRA (http://www.cost-terra.org/), commenced activities to identify the role and impact that emerging radio technologies will have on the development of the next generation of mobile communications. This article provides an overview of these selected key technologies and the way they will facilitate the realization of 5G, along with some of the research challenges that must be addressed in the coming years. It has to be noted that the technologies described in the paper do not cover all solutions that could be considered for future wireless systems, and one can find numerous excellent proposals in the vast literature, e.g., [3]. In our work, we address the solutions that are in line with the vision of 5G network we present in the following chapter. The remainder of this paper is organized as follows; as mentioned before—our vision for 5G systems is described in Sect. 2, enhanced modulation schemes for 5G are the topic of Sect. 3, and traffic offloading techniques are presented in Sect. 4 as the alternatives for complementing future cellular networks. The roles of cognitive radio, software-defined radio, and software-defined networking as 5G-enabling technologies are outlined in Sects. 5, 6, and 7, respectively. A discussion on 5G infrastructure is presented in Sect. 8 and we conclude in Sect. 9.

## Vision for 5G Systems

One central topic of debate is what the architecture of 5G systems will look like. The evolution from 1G to 4G was characterized mainly by a shift in the channel access method, i.e., FDMA $\rightarrow$ TDMA $\rightarrow$ CDMA $\rightarrow$ OFDMA, in conjunction with improved modulation and coding schemes. However, it is likely that 5G will not be a single air interface based on a single radio access technology (RAT) on the model of the previous generations. As modern communication systems approach the Shannon limit [19] and a wider range of devices demand wireless connectivity, some industry representatives envisage 5G as a “network of networks,” i.e., a heterogeneous system comprising a variety of air interfaces, protocols, frequency bands, access node classes, and network types [20]. In that vein, 5G is not really about the deployment of a completely new single-technology solution (something that will replace LTE, for example), but how existing technologies and spectrum-usage schemes can be better combined. If 5G reflects this prognosis, one of the major challenges will be the seamless integration of evolved versions of currently existing wireless technologies and complementary new communication networks.

### Expected Characteristics

The proliferation of mobile broadband services is expected to increase tremendously in the coming years. According to some forecasts [15, 20], telecommunication networks in 2020 will have to support more than one thousand times today’s mobile traffic volume. Currently, more than six billion wireless mobile terminals operate worldwide. Beyond 2020, the number of devices demanding wireless connectivity may reach fifty billion, comprising wireless sensors and machine communications [20]. Future mobile broadband systems are expected to provide users with the experience of radio access with “unlimited” performance, i.e., instantaneous delivery of large volumes of multimedia content over a highly stable wireless connection. Therefore, in addition to increased throughput, 5G systems must provide lower latencies, lower outage probability (i.e., better coverage), high bit rates over larger coverage areas, higher system spectral efficiency (data volume per area unit), higher versatility and scalability, lower infrastructure deployment costs, and higher reliability of communication links. Due to new types of connected devices (e.g., wireless sensors) in addition to current 2–4G mobile terminals, the requirements for data rate, latency, reliability, data payload, and power consumption will vary because of myriad different applications ranging from Internet of Things (IoT) to novel broadband services like ultra high resolution video and augmented reality [5]. Some applications may require peak data rates of several Gigabits per second and end-to-end latencies of a few milliseconds. For instance, wireless transmission of uncompressed lossless high definition (HD) video requires data rates of approximately 4 Gbps for 1080p resolution [32], which exceeds the capabilities of current wireless technologies. These top requirements must be set as goals for new research projects on 5G technologies; the European Commission has listed specific targets in the framework of the new program for research and innovation, Horizon 2020 (http://ec.europa.eu/research/horizon2020/), which commenced in 2014 and will run until 2020 [17]:

- Network architecture and protocols capable of at least a tenfold increase in frequency reuse.
- Technologies to avoid the spectrum crunch caused by explosive traffic growth.
- Efficient versatile radio access infrastructures allowing the seamless convergence of mobile and fixed networks.
- Real-time and flexible radio resource allocation (RRA) as a function of traffic distribution with quality of service (QoS) differentiation for multiple applications.
- Reduction of the network energy consumption by a factor of 10.
- End-to-end latency in the order of 1 ms.

### Radio Spectrum for 5G

Typically, each generation of mobile networks has been assigned new frequency bands and a wider spectral bandwidth per radio channel. The above vision for 5G systems will require a much more aggregated spectrum to enable flexible bandwidth scaling and expansion. To this end, additional harmonized frequency bands must be allocated. In order to increase frequency reuse, the spectrum shall be used on a RAT-neutral basis, ideally by applying cognitive radio principles to small and large cells [4]. The additional spectrum for 5G may include 100 MHz of bandwidth below 1 GHz to improve rural wireless broadband access and 500 MHz of the band between 1 and 5 GHz for enhanced high data rate capacity [41]. The core 3rd Generation Partnership Project (3GPP) frequency bands of 900, 1800, 2100, and 2600 MHz will be used for capacity upgrades in LTE-A and High Speed Packet Access (HSPA) networks. LTE-A will also rely on the 700 MHz band across the ITU Region 1 after the World Radio Communication Conference (WRC) in 2015 [73]. The long-term vision is the convergence of broadcast and broadband services in joint multimedia networks covering the ultra-high frequency (UHF) band below 700 MHz. Small cell deployments will play a vital role in high-capacity hotspots, and the spectrum for that could come from the 3500 MHz band, where as much as 400 MHz of bandwidth is used for fixed broadband wireless access and satellite services. The result is that up to 1.5 GHz of the spectrum can be made available by 2020; at least 1 GHz will be the traditional exclusive spectrum and the rest can be unlocked through the use of new spectrum-sharing techniques like the exploitation of TV White Spaces (TVWS) through the cognitive radio. TVWS are large portions of the VHF/UHF spectrum that are now available in geographic regions where the switchover from analog to digital TV has been completed (http://www.whitespacealliance.org/). Moreover, the LTE time-division duplexing (TDD) service in the 3.5 GHz band, called LTE-Hi, is also considered for hot-spot areas where coverage is not a demand, and the highest possible throughput is required. In addition, unlicensed bands such as 2.4, 5, and 60 GHz offer inexpensive spectrum options for traffic offloading. Especially the incorporation of 60 GHz with 7 GHz of available bandwidth is an opportunity for small cell backhaul solutions. For mobile wireless communications beyond 4G, i.e., beyond 3GPP Release 12 LTE-A, multiple spectrum opportunities can be found in the frequency, time, code, and angle domains [33, 75, 77]. Table 2 summarizes the frequency-domain opportunities foreseen for the cognitive radio that can be used for 5G.

### Ultra-Dense Radio Access Networks

A new concept foreseen in the context of 5G are Ultra-Dense Radio Access Networks (UDRANETs) [20]. UDRANETs are envisaged as low-power access nodes a few meters apart for indoor areas. The main goal of UDRANETs will be to provide an extremely high traffic capacity over highly-reliable short-range links. UDRANETs will likely operate in the 10–100 GHz frequency range, which has remained practically unused for commercial mobile wireless systems despite its potential to provide bandwidths of several hundreds of megahertz. New transmission and access technologies have to be developed and standardized for this type of networks, requiring spectrum allocation studies in millimeter waves (mmW) [8].

**Table 2:** Spectrum opportunities for 5G cognitive radios

Spectrum opportunity	Objective	Cognitive radio application
54–698 MHz	TV bands	Deployed under the carrier aggregation concept, i.e., using them as component carriers
2.7–2.9 GHz	Bandwidth scaling from 2.7 to 3.4 GHz for enhanced flexible spectrum usage	Not specified yet
3.4–3.6 GHz Band 43	A smooth exploitation of the Band 43 from WiMAX to LTE	Co-deployment on top of the macro cell layer
3.6–3.8 GHz	Contiguous carrier aggregation provision with the maximum 100 MHz bandwidth	Carrier aggregation application
3.8–4.2 GHz	Macro cell and small cell layers’ deployment, i.e., heterogeneous networks (HetNets)	Carrier aggregation within HetNets
60 GHz unlicensed band	Small cell backhaul deployment	Not specified yet

### Evolution Toward 5G

In our view, the evolution toward 5G will occur in two simultaneous phases: (1) the enhancement of current cellular networks compliant with 3GPP standards, and (2) the integration of evolved cellular networks with complementary wireless communication systems based on standards released by the IEEE and other standardization bodies. For the first evolution phase, enhanced modulation and coding schemes are the most obvious way to achieve a higher spectral efficiency at the physical (PHY) layer, as it has been done in the previous generations of mobile networks. Nevertheless, as the existing wireless technologies operate very close to the Shannon limit, little improvement is expected from this approach for point-to-point radio links [19]. Therefore, the carrier aggregation (CA) technology in LTE-A [44] will contribute significantly to higher data rates through the dynamic utilization of multiple continuous or non-continuous spectrum bands. For the second phase, we envisage that the integration of 3GPP-compliant cellular networks with other wireless communication systems will materialize through traffic offloading. Nevertheless, the feature that will differentiate 5G from previous mobile communication generations is the anticipated extensive use of cognitive radio, software-defined radio, and software-defined networking, i.e., 5G-enabling technologies, in combination with evolution technologies, i.e., new modulation schemes, CA, and traffic offloading. The following two sections explain the evolution technologies whereas the 5G-enabling technologies are discussed thereafter.

## Modulation Techniques for Higher Spectral Efficiency

Solid spectrum access methods and RRA algorithms will be crucial for efficient spectrum management in evolved RATs for 5G. The requirements regarding the permitted power levels transmitted in and outside the nominal band in the form of spectrum masks and acceptable interference power induced into neighboring systems have an impact on radio resource management (RRM). Such issues are of particular importance in the context of carrier-aggregation-like approaches (considered for LTE/LTE-A systems [44]), or in scenarios where the coexistence of various wireless systems in the same geographical area is allowed. The latter case has been widely discussed in the context of cognitive radio networks [39, 55, 65]. Based on the obvious observation that better spectrum utilization can be achieved when signals are closer to each other in the frequency domain, future 5G systems must attempt to minimize the allowed level of out-of-band (OOB) and spurious emissions. Moreover, in non-contiguous multicarrier transmission schemes where a small portion of subcarriers can be switched-off, e.g., non-contiguous orthogonal frequency-division multiplexing (NC-OFDM) [49, 72], the need for efficient and significant reduction of the power emitted in narrow spectrum gaps is particularly important. Such a scheme can be seen as a special case of CA for narrower sub-bands. For instance, let us consider the requirements defined by the Office of Communications in the United Kingdom [57] for the so-called White Space Devices (WSD). Although the values provided therein are strictly devoted to cognitive-radio-based solutions, it is wise to analyze these data and project them into the guidelines for 5G systems, where the reduction of the interference observed by neighboring users must be as high as possible. In [57] it is stated that the out-of-band equivalent isotropically radiated power (EIRP) spectral density, $P_{\mathrm{OOB}}$, expressed in terms of dBm per 100 kHz, must satisfy the following relation:

1 $$\begin{aligned} P_{\mathrm{OOB(dBm/100\,kHz)}}\le \max \left( P_{\mathrm{IB(dBm/8\,MHz)}}-A, 84\right) \end{aligned}$$

where $P_{\mathrm{IB}}$ is the in-block EIRP spectral density and *A* represents the adjacent frequency leakage ratio (AFLR), which varies from −74 to −54 dB, depending on the WSD class. These values have been adopted by the European Conference of Postal and Telecommunications Administrations-Electronic Communication Committee (CEPT ECC) and released in a dedicated report for WSD requirements, ECC Report 185 [18], and are proposed in the draft standard for WSD by the European Telecommunications Standards Institute (ETSI) [22]. Similar values have also been identified by the Federal Communications Commission (FCC) [26], where the fixed adjacent channel emission limit is −72.8 dB in a 100 kHz bandwidth, measured relative to the total in-band power over a 6 MHz bandwidth. One can notice that the requirements put on WSD are higher than the corresponding parameters in technical standards for current cellular systems [21]. Therefore, it can be stated that effective solutions that will guarantee a strong reduction of the power radiated outside the nominal frequency band will be applied not only for cognitive radio systems, but can—or rather should—be considered for the future 5G networks. Such an approach will lead to a lower interference inducement into adjacent channels and, in consequence, to a better utilization of the radio resources.

However, establishing such challenging restrictions on the interference induced to the adjacent band will, on the one hand, improve the spectral efficiency, but on the other, will entail the application of spectrally efficient modulation schemes and sophisticated transceiver front-ends. This is due to the fact that there are two main sources of unwanted power emission outside the nominal band: (1) the modulation process resulting in high sidelobes in OFDM systems, and (2) nonlinear phenomena appearing in the terminal front-end, e.g., the influence of high power amplifiers that possess non-linear processing characteristics; other electronic components should also be considered [31]. The latter strongly depends on the physical modules implemented in the device front-end. It has been shown that quite effective hardware solutions are already available [37], i.e., the level of OOB power, measured very close to the nominal band, can be reduced to the level of −100 dBm per 100 kHz, even for signals occupying the frequency band of 30 MHz. Therefore, it is more critical to focus on the first source of unwanted OOB emission and strive to improve the modulation processing.

**Fig. 1:**
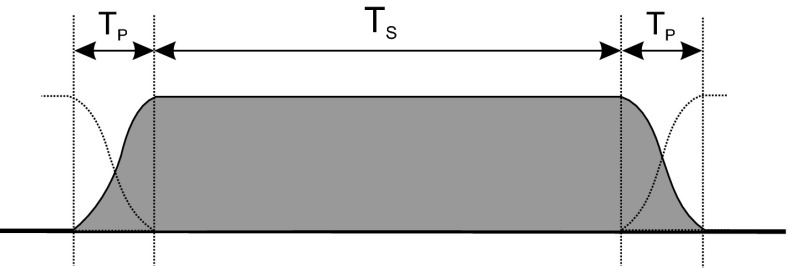
Illustration of OFDM symbol windowing; $T_S$ is the duration of the OFDM symbol (including cyclic prefix), and $T_P$ is the duration of the symbol pre- and post-fix

**Fig. 2:**
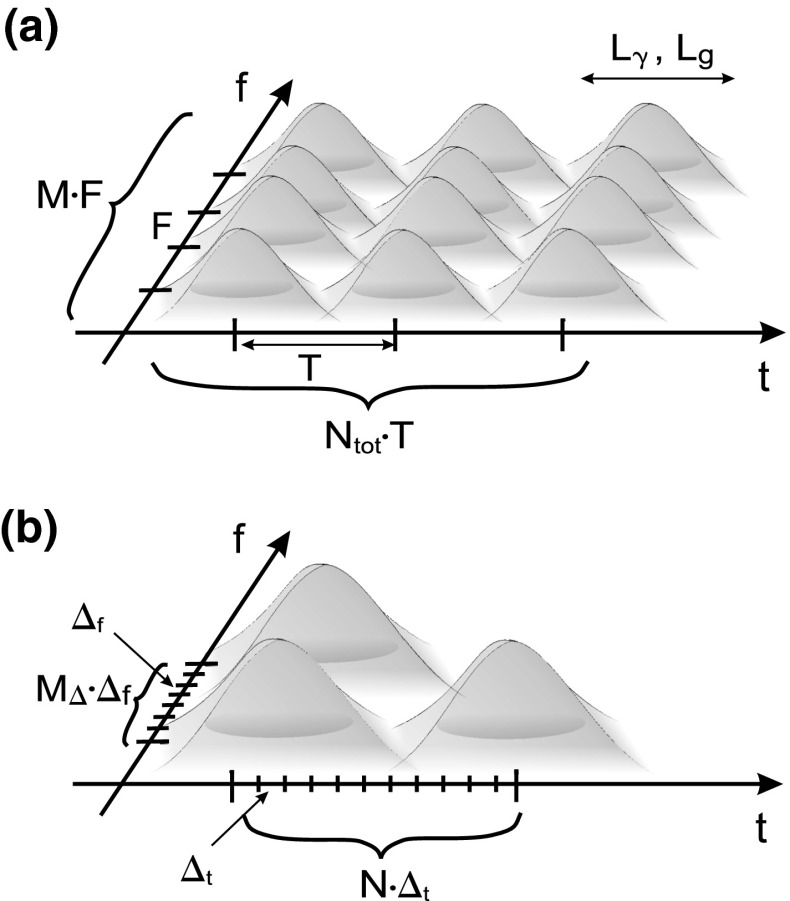
Time-frequency representation of the generic multicarrier frame

It is evident that the application of sophisticated PHY techniques can result in a better protection of the transmitted data, as well as the reduction of OOB emission levels, thereby ensuring a better exploitation of the spectrum and creating new degrees of freedom for its management. In order to guarantee a high spectral efficiency together with a relatively low complexity of the receiver structure, OFDM has been applied to many wireless systems, including 4G LTE. Nevertheless, one of the main drawbacks of OFDM is a high OOB power emission due to high sidelobes of the sinc-like transfer function of the transmit pulse. This undesirable phenomenon can be mitigated by extensive filtering and the use of sidelobe suppression algorithms, but even after applying such techniques, the amount of power radiated outside the nominal frequency band may remain high. To help address this issue, one technique involves extending the pulse duration to allow for smoother transitions between consecutive symbols. This is illustrated in Fig. 1 where the rectangular pulse is replaced by a square root raised cosine pulse with the time support of $T_s + 2T_p$ [67]. However, the cost associated with the extension of the duration of the time-domain symbol (the presence of a symbol prefix and postfix), is the reduction of the overall system efficiency (e.g., the rate expressed in bit/s/Hz/J). Another approach is to perform advanced filtering. However, it has to be taken into account that such signal processing improves the OOB reduction at the expense of some filtering distortions; an interesting analysis of the trade-off between interference and filtering distortions in OFDM systems can be found in [53]. Furthermore, the efficiency and the cost of various approaches are discussed in [24, 71]. These observations lead to the conclusion that new modulation processing technique should be applied; such techniques should guarantee a similar efficiency and robustness as OFDM, while outperforming the existing solutions in terms of OOB reduction. In this context, new modulation schemes have been promoted for mobile systems beyond 4G [30, 51], and cognitive radio solutions, e.g., the IEEE DySPAN-SC P1900.7 standard. Since rectangular (or almost rectangular) pulses, though simple in implementation and analysis, do not guarantee a low OOB emission, alternative solutions involve shaping the transmit and received pulses to result in a better energy concentration in the time-frequency plane. Let us first generalize multicarrier transmission by providing a generic signal description, and then indicate some of the most promising solutions that might be considered for the next generation of cellular networks. We denote *g*(*t*) and $\gamma (t)$ as the pair of transmit and receive pulses. Thus, the generic transmitted multicarrier signal *s*(*t*) and the estimation $\hat{c}_{m,n}$ of the user data, $c_{m,n}$ (e.g., Quadrature Amplitude Modulation (QAM) symbols), can be defined as:

2 $$\begin{aligned} s(t)=\sum _{m=0}^{M-1}\sum _{n\in {\mathbb {Z}}}c_{m,n}g_{m,n}(t) = \sum _{m=0}^{M-1}\sum _{n\in {\mathbb {Z}}}c_{m,n}g(t-nT)\exp \left( 2 \pi jmF(t-nT) \right) \end{aligned}$$

and

3 $$\begin{aligned} \hat{c}_{m,n}=\int _{-\infty }^{\infty } s(t)\gamma _{m,n}^* \,{\mathrm{d}}t = \int _{-\infty }^{\infty }\sum _{m'=0}^{M-1}\sum _{n'\in {\mathbb {Z}}}c_{m',n'}g_{m',n'}(t)\gamma _{m,n}^*\,{\mathrm{d}}t \end{aligned}$$

where *M* is the number of subcarriers, *T* is the time distance between consecutive pulses, *F* represents the distance between two neighboring pulses in the frequency domain, and $g_{m,n}(t)$ is the translated and modulated version of the original pulse, $g(t)$. Thus, the generic time-frequency representation of the transmit frame consisting of $M\cdot N_{\mathrm{tot}}$ pulses can be illustrated as in Fig. 2. In the OFDM case, the shape of both the transmit and received pulses is rectangular, the distance between the symbols in the time domain is set to $T_S$, and the distance between the adjacent subcarriers is reciprocal to the orthogonality time of the OFDM symbol. Relaxing these constraints, one can conclude that in the design process of the transmit-received pulse, various parameters have to be considered, such as the time and frequency support of the pulse, overlapping of pulses, and distance between pulses in both dimensions. Beside those parameters, also other criteria can be considered, such as the minimization of the minimum mean-square error (MMSE) of the assumed channel model [42], and others [9, 27, 46]. In the context of the reduction of OOB emission, the steepest possible power attenuation in the frequency domain is crucial, while maintaining the orthogonality or biorthogonality between pulses [27]. Intensive research has been done over the last decade in the area of efficient pulse shaping, and many newly proposed schemes offer clear advantages over purely rectangular shapes. Currently, among the various proposals that have been investigated, a class of pulses strictly devoted to filter bank-based multi-carrier (FBMC) systems seems quite promising [62]. Various pulse shapes have been considered for this modulation scheme [7, 23, 62, 64]. It has been shown that FBMC systems with isotropic orthogonal transfer algorithm (IOTA) filters can achieve an OOB attenuation at the level of −60 dB relative to the transmit power level in the nominal band. Moreover, good time-frequency localization of the pulses allows for removing the cyclic prefix typically applied in OFDM in order to combat the inter-symbol interference (ISI) problem. As a consequence, a higher spectral efficiency with a very low interference power induced to neighboring systems can be achieved at the expense of a slightly higher transceiver structure complexity. The FBMC transceiver could be implemented very effectively by means of inverse fast Fourier transform (IFFT) blocks followed by polyphase filters. However, despite its benefits, FBMC suffers from various other problems that still require attention, e.g., sensitivity to phase-noise or so-called intrinsic interference and its influence on signal detection [76]. A detailed analysis of the advantages and disadvantages of this solution has been presented in [62], and in extensive reports published within the framework of the PHYDYAS (http://www.ict-phydyas.org/) and EMPhAtiC (http://www.ict-emphatic.eu/) projects. In Table 3, we briefly summarize the main features of OFDM and FBMC signals in terms of their potential application in 5G systems.

**Table 3:** Comparison of OFDM and FBMC

Feature or parameter	OFDM	FBMC
Pulse shape	Pulse shape: rectangular (or almost rectangular in practical realizations due to various analogue filters)	Various shapes (e.g., IOTA, Gaussian, enhanced Gaussian pulse, Bellanger, etc.)
Cyclic prefix	Yes (up to 25 % of OFDM symbol duration)	No
Overlapping of pulses in time and frequency directions	Pulses do not overlap in time domain, orthogonality is preserved in frequency domain	Adjacent pulses overlap in both domains
AFLR	Poor (−13 dB for sinc-like shapes in frequency domain)	It can be kept very small (e.g., −60 dB for IOTA pulse)
Spectral efficiency	Limited by cyclic prefix	Limited by roll-off factor of the pulse
Complexity	It can be kept low; possible implementation with filter banks	It can be kept small, but always slightly higher than in OFDM; effective implementation with filter banks
Peak-to-average power ratio (PAPR)	High	High (it can be almost at the same level as for OFDM)

Another promising modulation scheme is NC-OFDM. In contrast to traditional OFDM signals, NC-OFDM enables a subset(s) of subcarriers to be switched off, allowing other signals to be transmitted in the vacated frequency bands. A low interference between neighboring signals can be achieved very efficiently by applying cancellation carriers (CCs) or windowing algorithms [49]. In such a case, the OOB emission can be minimized up to −60 dB in very narrow frequency gaps, where the power spectral density (PSD) of NC-OFDM signals with 320 subcarriers is presented. If implemented, it is conceivable that the above modulation schemes would serve as a supporting technology for RRA algorithms in 5G networks.

We will now outline the research activities focusing on generalized multicarrier schemes, where the constraints assumed in OFDM and even in FBMC schemes, are relaxed. The pair of the transmit and receive pulses, as well as the shape of the transmit frame, are designed in such a way that some specific predefined criteria are fulfilled. In [28, 38, 47, 59, 61, 63], the advantages of advanced waveforms are highlighted. In the context of 5G, which envisages the coexistence of various systems in close vicinity, together with the need for a better utilization of the frequency resources, the necessity of applying advanced waveforms is crucial. Although various proposals have been presented in the vast literature, FBMC and NC-OFDM schemes seem to be the most promising solutions at the current stage of development. The application of these waveforms will guarantee the fulfillment of the challenging OOB requirements (mentioned at the beginning of this section) that should be defined for 5G mobile terminals, as it has been done for WSD. At the same time, the spectral efficiency of these new modulation schemes will be kept at a very high level usually, outperforming the efficiency of today’s OFDM solutions. Such an observation is confirmed not only by the research community, but also by the recent standardization activities. For two-tier heterogeneous networks (HetNets), like macrocell/femtocell layouts, the recently proposed Vandermonde-subspace frequency division multiplexing (VFDM) [10] overlay spectrum sharing technique has the potential to provide a spectral efficiency increase of up to 1 bit/s/Hz in HetNets equipped with cognitive radio capabilities. Thus, new sophisticated modulation schemes are the key evolution technologies toward 5G networks.

## Mobile Traffic Offloading

Smartphones, tablets, and other mobile broadband devices generate unprecedentedly large amounts of traffic. Mobile operators are facing great challenges to serve such a tremendous traffic growth with the current cellular infrastructure. It is evident that mobile networks beyond 4G will need to implement sophisticated traffic offloading strategies [2] beside the traditional network scaling of deploying more base stations per area. Traffic offloading consists in using complementary radio access networks to deliver data originally intended for mobile cellular systems, thereby decreasing the congestion on each individual radio link and respective backbone connection. Traffic offloading encompasses multiple solutions, which can be classified as overlay (sharing the same spectrum with the cellular access network) and non-overlay solutions; some of them are discussed below.

### Cognitive Femtocells

Femtocell traffic offloading is based on the deployment of small, low-power cellular base stations at home or in other indoor areas, backhauled to the cellular core network by a conventional wired network [35]. Among several benefits of this solution is the fact that femtocells can handle both voice and data traffic with QoS assurance. However, the use of the same spectrum as overlaying macrocells complicates the search for available channels for femtocells in highly congested areas. Hence, intelligent interference management using cognitive radio and RRA coordination must be applied, especially in deployment scenarios where end users decide about the locations of the femtocells, e.g., uncoordinated home eNB. The highly-unpredictable interference with macrocells requires an opportunistic spectrum access within a hierarchical overlay system as follows: first, a sensing procedure at the macrocell level provides the knowledge about spectrum opportunities, which are subsequently exploited by the femtocells [36, 41, 66, 69].

### Wi-Fi and White-Fi

**Fig. 3:**
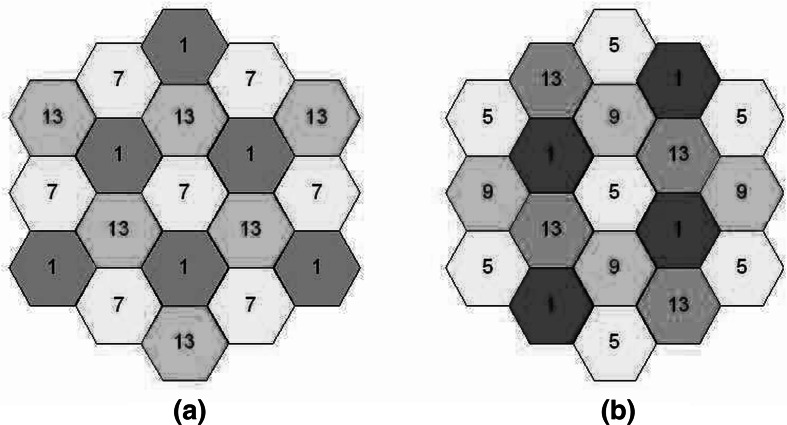
Indoor multicell Wi-Fi layout in which a BSS transmits VoIP traffic in the central cell with a radius equal to 30 m. **a** Configuration with 3 non-overlapping channels (13 overlapping channels are allocated in Europe on the 2.4 GHz band), and **b** configuration with 4 slightly overlapped channels

**Fig. 4:**
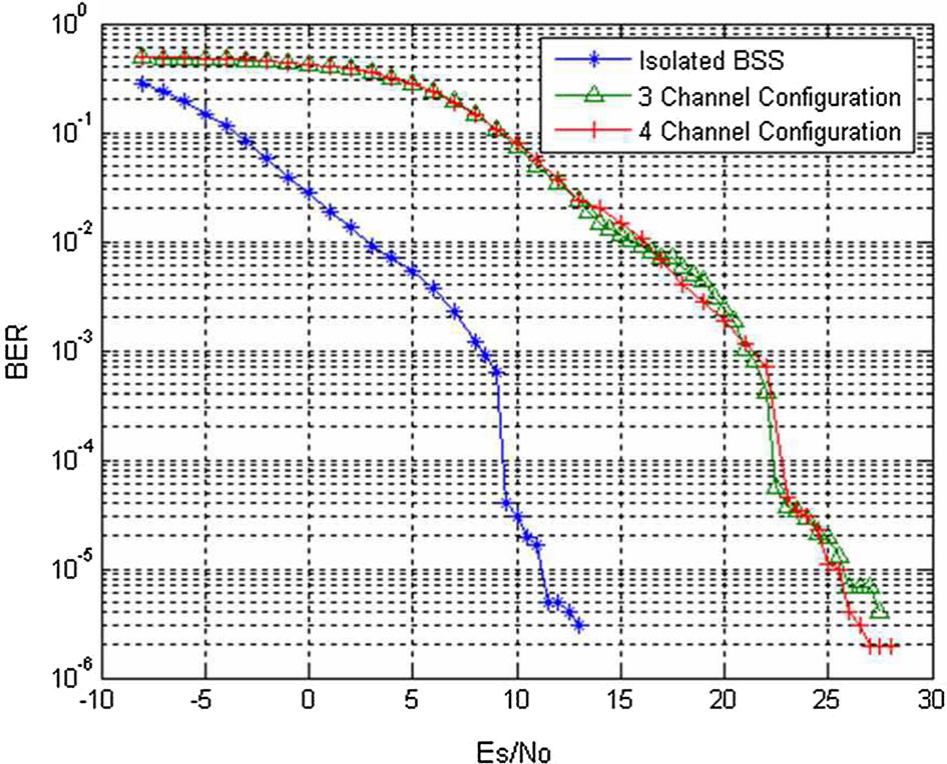
Bit-error-rate (BER) performance of the BSS VoIP transmission subjected to interference from surrounding cells in the layouts depicted in Fig. 3. In the isolated BSS case no interference from surrounding cells was present

Non-overlay traffic offloading via Wi-Fi networks has already been implemented by some cellular operators. Basically, when a mobile terminal is in the vicinity of a Wi-Fi hotspot, data traffic routing is switched to exploit its radio interface. This solution is attractive as it allows access to a free, unlicensed spectrum, thereby reducing the unnecessary congestion in expensive, licensed frequency bands. Strategic alliances between cellular operators and Internet service providers (ISPs) need to be established. Nevertheless, the Wi-Fi medium access control (MAC) protocol is not well-suited for a heavy traffic load and does not provide QoS differentiation. Consequently, this solution is currently implemented for best-effort traffic only, whereas voice services are still delivered via the cellular core network. One major challenge is to increase the system spectral efficiency by allowing more concurrent users on Wi-Fi networks, ideally for both best-effort and voice traffic. A possible way of achieving this is to implement Wi-Fi frequency reuse plans with slightly overlapping channels (Fig. 3). In a case study [12], computer simulations of an indoor IEEE 802.11b basic service set (BSS) subject to interference from surrounding cells demonstrated that transmissions of voice over Internet protocol (VoIP) are not significantly degraded in the 2.4 GHz band by the resulting co-channel interference (Fig. 4). For full VoIP offloading capabilities, however, the QoS mechanism already available in the IEEE 802.11 standards should be implemented. A more recent variant of this offloading approach is White-Fi, which aims at using the TVWS spectrum through cognitive radio for a transmission with the conventional Wi-Fi technology. In 2013, this solution was standardized in IEEE 802.11af.

### Other Offloading Solutions

WiMAX can be considered as an offloading alternative, but it is more suitable as the backhaul for large-scale Wi-Fi networks. Furthermore, 3GPP compliant cellular networks have not considered interoperation with WiMAX so far; therefore, additional standardization would be required.

When communicating users are geographically close to each other, device-to-device (D2D) communication [16] can also help diminish traffic congestion on the cellular core network. D2D communication is an underlay to cellular networks in licensed frequency bands, contrasting with mobile ad-hoc networks (MANETs), which operate similarly but in the unlicensed spectrum.

**Fig. 5:**
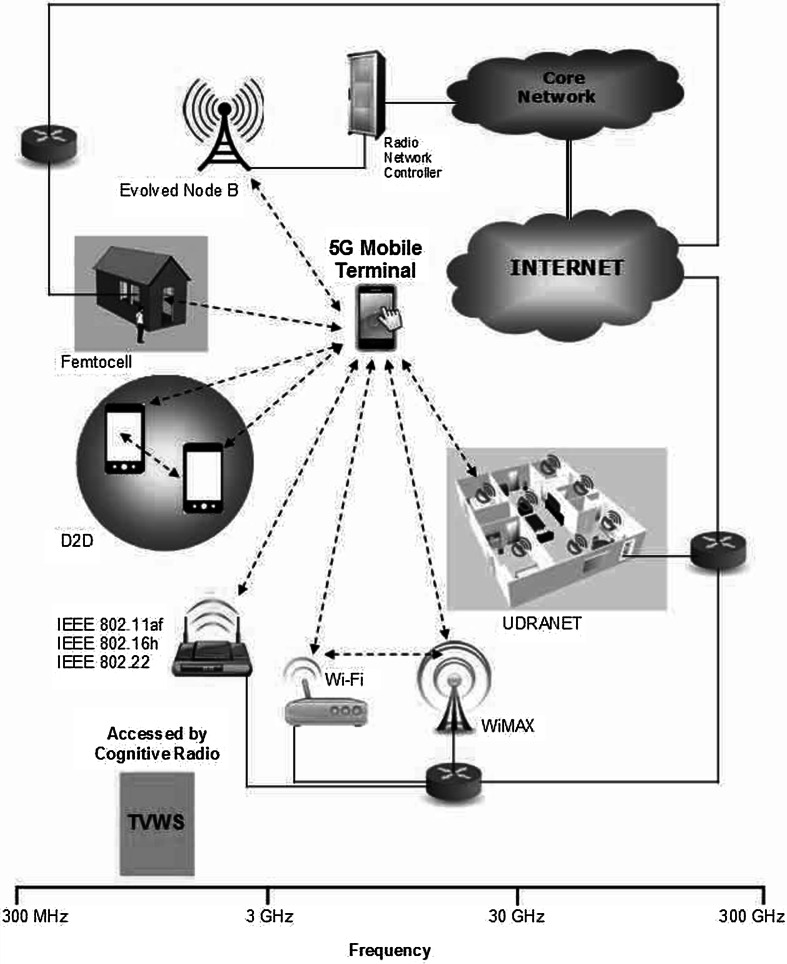
Traffic offloading approaches for 5G systems

The next generation of cellular systems could leverage offloading opportunities created by the integration of the aforementioned solutions and others that may be developed in the future. These solutions can be further extended through the use of cognitive radio, an approach we refer to as cognitive mobile-traffic offloading (CMTO). For instance, an outdoor mesh network of Wi-Fi and White-Fi hotspots backhauled to the cellular network through WiMAX links and/or wired broadband access can serve as traffic offloading alternatives in combination with femtocells and D2D links (Fig. 5).

### Research Challenges

Traffic offloading solutions will allow the virtual expansion of the spectrum allocated to cellular networks, thereby increasing their capacity. However, important issues, like user authentication, network security, and service pricing [2] must be addressed first. The emerging software-defined networking technology has the potential to solve some of these problems, since this approach provides a radio network controller (RNC) with a real-time updated view of the state of each link and device in the network, as it will be explained later. From the user perspective, service continuity and same level of connectivity must be ensured independently of the access network [add: (cf. Sect. 6)].

UDRANETs will perform the role of traffic offloading alternatives for extremely high data rate applications, but the main challenge here is to produce low-cost mobile terminals that can operate in super-high frequency (SHF) and extremely-high frequency (EHF) bands. On the other hand, the use of mmW enables packing more antennas into a terminal, thereby facilitating the implementation of the massive multiple-input multiple-output (M-MIMO) for an improved throughput and a lower latency [50]. In combination with multiuser MIMO (MU-MIMO), cellular networks can benefit from a significant capacity increase. These topics represent many opportunities for research and innovation.

## Cognitive Radio

Cognitive radio (CR) has long been considered an enabling technology for the next generation of mobile communications [6]. The CR paradigm proposes an opportunistic utilization of the underused parts of licensed frequency bands, i.e., spectrum holes [68], by unlicensed (secondary) users and/or the efficient sharing of the licensed-exempt spectrum. For this sake, cognitive mobile terminals must acquire accurate real-time knowledge on transmission opportunities through RF spectrum scanning to identify the unoccupied radio channels/bands within the time-frequency resources table. Standards like IEEE 802.11af, IEEE 802.16h (cognitive WiMAX), and IEEE 802.22 are aimed at using CR techniques to enable sharing of the TVWS spectrum on a non-interfering basis with improved coexistence mechanisms.

The CR technology is now at an advanced stage and real-world trials and evaluations are being carried out. As an example, an experimental trial of CR and the dynamic spectrum access technology using the TVWS spectrum is being carried out in Ireland. This Irish research testbed is assessing the feasibility of taking an opportunistic spectrum usage approach in the 700 MHz band to establish an 18.3 km TVWS link connecting the Trinity College Dublin in Dublin city center to Intel Labs Europe (Ireland) based in Leixlip, Co. Kildare. Demonstrating the spectrum agility and the ability to avoid interference to incumbents in order to provide a robust link suitable for the support of new services is the key objective of this trial [56]. In future 5G systems, CR will be a key component to ensure interference coordination in 3GPP-compliant overlay networks as well as in different traffic offloading techniques. The 3GPP LTE standard has taken provisions to facilitate interference mitigation in overlay networks. Enhanced Intercell Interference Coordination (eICIC) was introduced in 3GPP Release 10 LTE-A to tackle interference issues in HetNets. In this approach, Almost-Blank-Subframes (ABSs) are introduced, which are mostly control channel frames with a very low power. A macrocell can configure ABSs, so that mobile terminals in small cells can send their data within ABSs to avoid interference with overlay macrocells. The use of CR can improve this approach resulting in a more efficient utilization of the radio resources with an even better interference coordination for two-tier HetNets, which ultimately leads to an enhanced system capacity and coverage [13]. CA for HetNets developed in compliance with 3GPP Release 12 LTE-A will introduce new CR-wise techniques for efficient spectrum allocation in heterogeneous wideband fading environments.

### Research Challenges

In addition to CA in LTE-A systems, the CA of multiple and heterogeneous RATs (h-RATs) will also be a key feature of 5G communications, which will require CR application at all layers. Some of the associated challenges will be addressed by the recently established EU Project “Spectrum Overlay through Aggregation of heterogeneous Dispersed bands,” SOLDER (see Table 1). The objectives of the project are: a non-continuous aggregation of heterogeneous bands, flexible multicarrier modulation, efficient MAC layer design and the aggregation of heterogeneous resources. Furthermore, although a number of CR solutions for underlay femtocells have been recently proposed, fewer CR approaches for underlay D2D communication are available in the literature. Research in this area is therefore required.

## Software Defined Radio

The future 5G network infrastructure must have a sufficiently wide flexibility in order to cover different radio frequency (RF) parameter settings in a dynamic and adaptive way to allow efficient spectrum management. Reconfigurable platforms based on software-defined radio (SDR) will facilitate the dynamic air interface reconfiguration of the network nodes by software modifications, reflecting current traffic demands. The flexibility of the RF chains must be further reflected in the baseband processing capabilities, where the down-converted RF signals are to be processed. As 5G systems will need to exploit underutilized frequency bands to avoid the foreseen spectrum crunch, CR implementation on SDR platforms should consider the cooperation and interoperation of multiple radio technologies, e.g., through common radio resource management (CRRM). This implies that a reconfigurable platform must be capable to operate at different power levels, channel bandwidths and frequencies, modulation and coding schemes, modifying transmission parameters and characteristics according to the particular constraints of the radio technology standards in use; these constraints include unwanted emission in the operating band, AFLR, or intermodulation products. Promising SDR development initiatives include the GNU Radio (http://gnuradio.org/), an open-source software development toolkit for SDR implementation on various programmable platforms like the Universal Software Radio Peripheral (USRP) boards (http://www.ettus.com/), and the Open Base Transceiver Station (OpenBTS), which was recently used to demonstrate the implementation of a software-based Global System for Mobile Communications (GSM) base station on the Raspberry PI hardware platform (http://openbts.org/).

Looking at recent developments for LTE-A, the CA feature [70] with its maximum configuration of $5\times 20$ MHz radio channels is a good starting point for future research on the incorporation of cognition, to further improve the flexibility of radio resources utilization. Nevertheless, the RF chains bandwidth and their tuning are considered here as a bottleneck, especially when a variety of different frequency bands are used for cellular networks in different markets. The multi-standard radio base station (MSR-BS) addresses these issues in the recent releases of 3GPP networks, enabling a single radio unit in the base station to simultaneously operate with multiple RATs at the same time without harming each other with unwanted RF emissions.

### Research Challenges

Despite a significant progress in this field, further research is required for the practical implementation of SDR-based reconfigurable systems for 5G, such as an SDR-based MSR-BS. To fulfill the vision for 5G systems, the user equipment (UE) also needs to be fully reconfigurable [45] and able to tune the RF chain(s) to different aggregated radio channels and h-RATs, as dictated by the scheduler on the network side. Cognition aspects will have to be incorporated into self-organizing network (SON) features in future 3GPP standards releases, extending the dynamic network selection procedures. The major challenge in these cases is the design of the radio front-end to properly and efficiently handle the large operation bandwidth, from 700 MHz to 3.5 GHz. Furthermore, following the market trends, mobile terminals must be limited in size, power consumption, and cost.

## Software Defined Networking

At the current stage of development, both CR and SDR technologies do not involve the control of the cellular core network. Until now, no coordination of traffic flow is possible at the core network, e.g., a UE cannot receive multiple different traffic flows from different eNBs simultaneously to increase the data rate. The revolutionary concept of software-defined networking (SDN) aims at providing a coordinator that has a global view of the network infrastructure, thereby facilitating a number of networking functionalities. In essence, the SDN concept consists in decoupling the forwarding plane and the control plane [48]. In this approach, the core devices, e.g., routers, do not make decisions on how and where to forward a given data packet; instead, the decision is taken by a central coordinator referred to as the controller. One of the protocols in charge of the communication between the controller and user devices is OpenFlow [54]. Data packets passing through interconnection devices (switches, routers, etc.) are classified into flows to make per-flow forwarding decisions. A flow is defined by a set of matching rules in 12 distinctive fields of a typical Ethernet/IP/UDP header (L2 and L3 addresses, virtual LAN (VLAN) information, ports, etc.). Each time a data packet affiliated to a specific flow enters a device, a counter is updated at the controller. This enables the controller to have a global view of the status of each network component. Therefore, the controller can make the decision to forward the traffic through a less congested route, or use a radio channel that momentarily experiences a good condition to forward the data packet to the end user [11]. SDN aims to support a much better integration of all the existing wireless networks (Wi-Fi, 2–4G, etc.) [74]. It would be possible to perform a seamless handover, not only within the same technology as already exists, but also across h-RATs. This feature will enable the offloading approaches described in Sect. 4. Moreover, SDN will greatly facilitate the management of chaotic deployments of a large number of small cells in LTE systems [34]. The ultimate goal of SDN is to completely abstract the forwarding plane to enable “network programmability” where the infrastructure is seen as a service that can be used by different applications above an intermediate layer referred to as the Network Operating System (NOS). This is the basis for the concept of cloud networks. In the context of 5G, we can refer to this approach as cloud-RAN or RAN as a Service (RANaaS).

### Research Challenges

SDN entails some interesting drawbacks and challenges. For instance, security threats could be more severe in SDN. An attack on a controller could compromise the connection of a large coverage area. In the case of an inter-ISP handover, the collaboration between multiple controllers affiliated to different ISPs is required. However, to what extent and how to promote this kind of collaboration requires careful analysis, as it implies the exchange of network status information that most operators prefer to keep confidential for business purposes. More generally, the optimal balance between centralized and decentralized management needs to be established. It is clear that a fully distributed scheme does not allow the optimal utilization of the spectrum, since this cannot be achieved with partial or local knowledge; but on the other hand, it is not realistic to imagine a controller in charge of a very large area, since this could introduce too much latency in terms of answer-to-flow matching rules and device feedback processing.

**Fig. 6:**
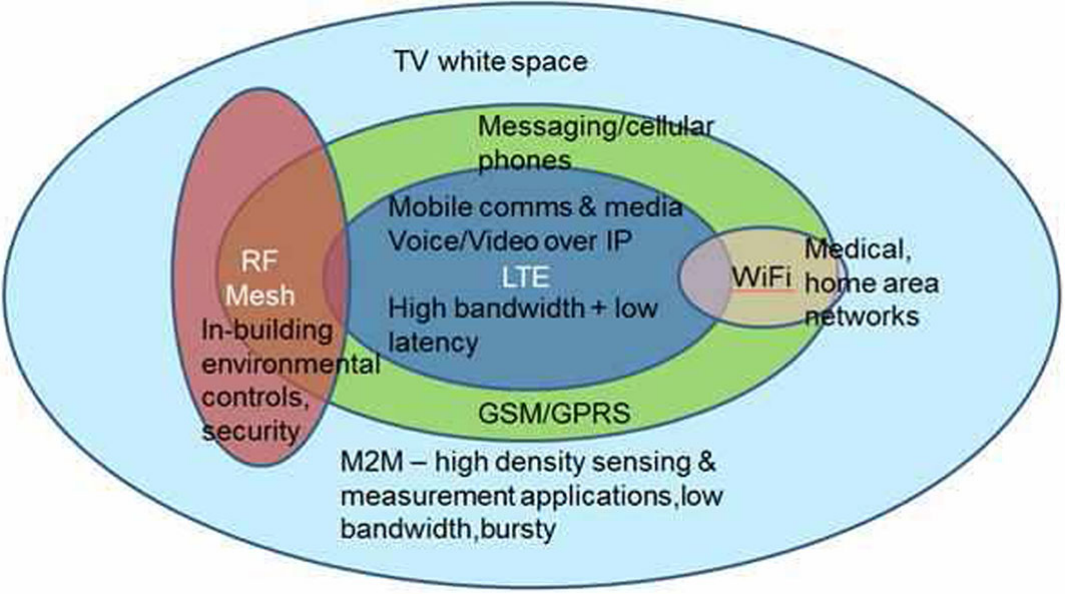
5G as a blended infrastructure encompassing the range of cellular, local and personal area network, and short range technologies and their associated regulatory approaches, e.g., exclusive, light, licence-exempt, and shared spectrum models

## Blended Infrastructure

5G systems will leverage the availability and variety of the deployed infrastructure to better address the need for capacity, coverage, and high quality of service instead of relying on a single technology, topology, or regulatory approach [29]. In other words, unlike 4G, which involves exclusively-licensed LTE in a predominantly cellular-based topology, 5G will focus on knitting together cellular, local and personal area networks, short range device technologies and topologies. This concept also extends to the regulatory domain; 5G communications will encompass a range of exclusive, light-licensing, license-exempt, and shared spectrum licensing schemes. Through this approach, the flow of data can be more efficiently abstracted from the physical infrastructure and regulatory approach. This approach is likely to increase in dominance over the coming years due to two main reasons: (1) it is unlikely that a single technology/standard will be able to provide a sufficient capacity and coverage to accommodate all market sectors; (2) networks seeking to maximize the utility of existing infrastructure in a bid to increase the return on capital investments will continue to pursue new avenues for offloading, data aggregation, active sharing, and licensed shared access options. As a result, they will position themselves as parts of a blended infrastructure, instead of a standalone network, as in the majority of operator models today. An example of how this blended infrastructure may be implemented is illustrated in Fig. 6. In this example, RF mesh and shared spectrum technologies, e.g., TVWS, complement a core cellular network technology, e.g., LTE, for capacity and high data-rate delivery, and 2–3G cellular technologies for coverage. Wi-Fi will continue to play an important part in home area networks and as part of operator data-offloading strategies. Short range devices, e.g., ZigBee and Z-Wave will dominate the in-building control sector. 5G will better leverage the advantages stemming from a blend of technology and spectrum-usage options. This could lead to usage scenarios in which the communications flow could better suit customers’ priorities, e.g., the requirement for a high QoS and willingness to pay extra for the service, versus low cost communication requirement and acceptance of a lower QoS or higher latency services.

### Societal Impact of 5G

From the societal perspective, 5G networks have the potential to improve the mobile broadband access in rural areas. The capital expenditure for deploying a large number of base stations and the low average revenue per user (ARPU) [25] has delayed the complete coverage of rural environments. By using TVWS and traffic offloading solutions, the deployment of 5G networks in rural areas will be possible at a lower cost thanks to more favorable propagation conditions in the VHF/UHF spectrum that directly translate into fewer base stations.

## Conclusions

In the quest for higher data rates and lower latencies beyond 4G network capabilities, the next generation of wireless mobile communications has to adopt revolutionary ways of using the radio spectrum. In addition to better ways of exploiting the already allocated frequency bands through enhanced modulation schemes and traditional network scaling, an opportunistic access to the underused spectrum through cognitive radio will further increase the systems’ capacity. Moreover, different offloading techniques will contribute to an “unlimited” access to large amounts of multimedia data anywhere and anytime. The software-defined radio technology will enable reconfigurable platforms for the implementation of the cognitive radio paradigm in underlay cellular networks and traffic offloading solutions, whereas software-defined networking will facilitate the programmable operation of the core network. The evolution towards 5G, as outlined in this paper, opens new research problems. Although we have briefly surveyed the key research projects recently launched in Europe, efforts to develop 5G technologies are being carried out worldwide. Further standardization for cooperation and interoperability between different radio access technologies will be needed, too. With this paper, we intend to motivate the search for innovative solutions to all these challenges.

## Footnotes

1 COST stands for “European Cooperation in Science and Technology.” COST Actions are intergovernmental frameworks to allow the coordination of nationally-funded research on a European level. More information at http://www.cost.eu/.

## References

[1] Agyapong P, Iwamura M, Staehle D, Kiess W, Benjebbour A (2014). Design considerations for a 5G network architecture. IEEE Communications Magazine.

[2] Aijaz A, Aghvami H, Amani M (2013). A survey on mobile data offloading: Technical and business perspectives. IEEE Wireless Communications.

[3] Akyildiz IF, Gutierrez-Estevez DM, Balakrishnan R, Chavarria-Reyes E (2014). LTE-advanced and the evolution to beyond 4G (B4G) systems. Physical Communication.

[4] Akyildiz IF, Lee WY, Vuran MC, Mohanty S (2006). NeXt generation/dynamic spectrum access/cognitive radio wireless networks: A survey. Computer Networks.

[5] Akyildiz IF, Melodia T, Chowdury KR (2007). Wireless multimedia sensor networks: A survey. IEEE Wireless Communications.

[6] Badoi CI, Prasad N, Croitoru V, Prasad R (2011). 5G based on cognitive radio. Wireless Personal Communications.

[7] Bellanger, M. (2001). Specification and design of a prototype filter for filter bank based multicarrier transmission. In *Proceedings of the IEEE international conferences acoustics, speech, and signal processing (ICASSP), Salt Lake City, UT* (pp. 2417–2420).

[8] Bleicher, A. (2013). *Millimeter waves may be the future of 5G phones*. http://spectrum.ieee.org/telecom/wireless/millimeter-waves-may-be-the-future-of-5g-phones.

[9] Bolcskei, H., Duhamel, P., & Hleiss, R. (1999). Design of pulse shaping OFDM/OQAM systems for high data-rate transmission over wireless channels. In *Proceedings of the IEEE international conference on communications (ICC), Vancouver, BC* (pp. 559–564).

[10] Cardoso LS, Kobayashi M, Cavalcanti FRP, Debbah M (2013). Vandermonde-subspace frequency division multiplexing for two-tiered cognitive radio networks. IEEE Transactions on Communications.

[11] Chaudet, C., & Haddad, Y. (2013). Wireless software defined networks: Challenges and opportunities. In *Proceedings of the international IEEE conference on microwaves, communications, antennas and electronics (COMCAS), Tel-Aviv, Israel* (pp. 21–23).

[12] Chavez-Santiago, R., Haddad, Y., Lyandres, V., & Balasingham, I. (2015). Voip transmission in Wi-Fi networks with partially-overlapped channels. In *Proceedings of IEEE wireless communications and networking conference (WCNC), New Orleans, USA*.

[13] Cheng SM, Lien SY, Chu FS, Chen KC (2011). On exploiting cognitive radio to mitigate interference in macro/femto heterogeneous networks. IEEE Wireless Communications.

[14] Chih-Lin I, Rowell C, Han S, Xu Z, Li G, Pan Z (2014). Toward green and soft: A 5G perspective. IEEE Communications Magazine.

[15] Cisco. (2013). *Cisco visual networking index: Global mobile data traffic forecast update*. White paper by Cisco. http://www.cisco.com/en/US/solutions/collateral/ns341/ns525/ns537/ns705/ns827/white_paper_c11-520862.pdf.

[16] Doppler K, Rinne M, Wijting C, Ribeiro CB, Hugl K (2009). Device-to-device communication as an underlay to LTE-advanced networks. IEEE Communications Magazine.

[17] European Commission. (2013). *Horizon 2020-Work Program 2014–2015. Leadership in enabling and industrial technologies information and communication technologies*. https://ec.europa.eu/digital-agenda/sites/digital-agenda/files/h2020 LEIT-ICT WP.

[18] Electronic Communication Committee. (2013). *Complementary report to ECC report*. http://www.erodocdb.dk/Docs/doc98/official/pdf/ECCREP185.pdf.

[19] Edwards, C. (2013). 5G mobile model challenged by ‘spectrum crunch’. *E&T Magazine, 8*(8). http://eandt.theiet.org/magazine/2013/08/5g-searches-for-formula.cfm.

[20] Ericsson. (2013). *5G radio access*. White paper. http://www.ericsson.com/res/docs/whitepapers/wp-5g.pdf.

[21] ETSI. (2012). LTE; evolved universal terrestrial radio access (E-UTRA); user equipment (UE) radio transmission and reception. ETSI Technical Specification ETSI TS 136 101 V9.12.0.

[22] ETSI. (2013). White space devices (WSD); wireless access systems operating in the 470 MHz to 790 MHz frequency band; harmonized EN covering the essential requirements of article 3.2 of the R&TTE Directive. Standard Draft ETSI EN 301 598 V1.0.0.

[23] Farhang-Boroujeny B (2011). OFDM versus filter bank multicarrier. Signal Processing Magazine.

[24] Farhang-Boroujeny B, Kempter R (2008). Multicarrier communication techniques for spectrum sensing and communication in cognitive radios. IEEE Communications Magazine.

[25] Fehske A, Fettweis G, Malmodin J, Biczok G (2011). The global footprint of mobile communications: The ecological and economic perspective. IEEE Communications Magazine.

[26] Federal Communications Commission. (2012). *Third memorandum opinion and order*. http://transition.fcc.gov/Daily_Releases/Daily_Business/2012/db0405/FCC-12-36A1.pdf.

[27] Feichtinger HG, Strohmer T (1998). Gabor analysis and algorithms: Theory and applications (Applied and Numerical Harmonic Analysis).

[28] Fettweis, G., Krondorf, M., & Bittner, S. (2009). GFDM-generalized frequency division multiplexing. In *Proceedings of the IEEE 69th vehicular technology conference (VTC 2009-Spring), Barcelona, Spain* (pp. 1–4).

[29] Forum, W. I. (2013). Wireless innovation forums comments to ITU WP-5A regarding the working document towards a preliminary draft new report ITU-R [LMS CRS2] cognitive radio systems [(CRS) applications] in the land mobile service. http://groups.winnforum.org/d/do/6929.

[30] Gameiro, A., (Ed.) (2010). *Radio environment models. Deliverable of the QoSMOS project*. http://www.ict-qosmos.eu/fileadmin/documents/Dissemination/Deliverables/files/DelPub/QoSMOS_WP3_D31.pdf.

[31] Gharaibeh KM (2012). Nonlinear distortion in wireless systems: Modeling and simulation with MATLAB.

[32] Gilbert JM, Doan CH, Emami S, Shung CB (2008). A 4-Gbps uncompressed wireless HD A/v transceiver chipset. IEEE Micro.

[33] GmbH, N. R. (2013).* GPP LTE—A standardisation in Release 12 and beyond*. White paper. http://www.nomor.de/uploads/fd/24/fd24709a64bc490a083a8eba6d3cc2cb/NoMoR_LTE-A_Rel12_and_Beyond_2013-01.pdf.

[34] Gudipati, A., Perry, D., Li, L. E., & Katti, S. (2013). SoftRAN: Software defined radio access network. In *Proceedings of the 2nd ACM SIGCOMM workshop on hot topics in software defined networking (HotSDN), Hong Kong, China* (pp. 25–30).

[35] Haddad, Y., & Porrat, D. (2009). Femtocell: Opportunities and challenges of the home cellular base station for the 3G. In *IADIS international conference wireless applications and computing (WAC)*.

[36] Haddad, Y., & Porrat, D. (2010). A two-tier frequency reuse scheme. In *Personal, indoor and mobile radio communications workshops (PIMRC Workshops), 2010 IEEE 21st international symposium on* (pp. 203–207). doi:10.1109/PIMRCW.2010.5670362.

[37] Hammi O, Kwan A, Ghannouchi FM (2013). Bandwidth and power scalable digital predistorter for compensating dynamic distortions in RF power amplifiers. IEEE Transactions on Broadcasting.

[38] Han FM, Zhang XD (2009). Wireless multicarrier digital transmission via Weyl–Heisenberg frames over time-frequency dispersive channels. IEEE Transactions on Communications.

[39] Haykin S (2005). Cognitive radio: Brain empowered wireless communications. IEEE Journal on Selected Areas in Communications.

[40] Hossain E, Rasti M, Tabassum H, Abdelnasser A (2014). Evolution toward 5G multi-tier cellular wireless networks: An interference management perspective. IEEE Wireless Communications.

[41] Huang L, Zhu G, Du X (2013). Cognitive femtocell networks: An opportunistic spectrum access for future indoor wireless coverage. IEEE Wireless Communincations.

[42] Hunziker, T., Ju, Z., & Dahlhaus, D. (2007). Efficient channel description in time-frequency domain with application to flexible radio. In *Proceedings of the15th European signal processing conference (EUSIPCO), Poznan, Poland* (pp. 866–870).

[43] Imran A, Zoha A (2014). Challenges in 5G: How to empower son with big data for enabling 5G. IEEE Network.

[44] Iwamura M, Etemad K, Mo-Han F, Nory R, Love R (2010). Carrier aggregation framework in 3GPP LTE-advanced [WiMAX/lte Update]. IEEE Communications Magazine.

[45] Janevski, T. (2009). 5G Mobile phone concept. In *Proceedings of the 6th IEEE consumer communications and networking conference, Las Vegas, NV* (pp. 1–2).

[46] Jung P, Wunder G (2007). The WSSUS pulse design problem in multicarrier transmission. IEEE Transactions on Communications.

[47] Kozek W, Molisch AF (1998). Nonorthogonal pulseshapes for multicarrier communications in doubly dispersive channels. IEEE Journal on Selected Areas in Communications.

[48] Kreutz, D., Ramos, F. M. V., Veríssimo, P., Rothenberg, C. E., Azodolmolky, S., & Uhlig, S. (2014). *Software-defined networking: A comprehensive survey.* CoRR abs/1406.0440.

[49] Kryszkiewicz P, Bogucka H (2013). Out-of-band power reduction in NC-OFDM with optimized cancellation carriers selection. IEEE Communications Letters.

[50] Larsson, E., Edfors, O., Tufvesson, F., & Marzetta, T. (2014). Massive MIMO for next generation wireless systems. *IEEE Communications Magazine*, *52*(2), 186–195.

[51] Lhetkangas, E., Lin, H., (Eds.) (2013). Deliverable D2.1. Requirement analysis and design approaches for 5G air interface. METIS Deliverable [online]. https://www.metis2020.com/wp-content/uploads/deliverables/METIS_D2.1_v1.pdf.

[52] Li, X., Gani, A., Salle, R., & Zakaria, O. (2009). The future of mobile wireless communication networks. In *Proceedings of the international conference on Communication software and networks, Chengdu, China* (pp. 554–557).

[53] Maliatsos K, Adamis A, Kanatas AG (2013). Interference versus filtering distortion trade-offs in OFDM-based cognitive radios. Transactions on Emerging Telecommunications Technologies.

[54] McKeown N, Anderson T, Balakrishnan H, Parulkar G, Peterson L, Rexford J (2008). OpenFlow: Enabling innovation in campus networks. ACM SIGCOMM Computer Communication Review.

[55] Mitola, J. (2000). *Cognitive radio—An integrated agent architecture for software defined radio*. Ph.D. thesis. Royal Institute of Technology (KTH): Sweden.

[56] Nolan, K. E., Kelly, M. Y., Forde, T., & Doyle, L. E. (2015). Development of a white space spectrum wireless interconnector. In *Proceedings of the 10th international conference on cognitive radio oriented wireless networks (CROWNCOM), Doha, Qatar*. April 21–23, 2015.

[57] Ofcom. (2012). *TV white spaces—A consultation on white space device requirements*. http://stakeholders.ofcom.org.uk/binaries/consultations/whitespaces/summary/condoc.pdf.

[58] Osseiran A, Boccardi F, Braun V, Kusume K, Marsch P, Maternia M (2014). Scenarios for 5G mobile and wireless communications: The vision of the metis project. IEEE Communications Magazine.

[59] Pagadarai S, Kliks A, Bogucka H, Wyglinski AM (2011). Non-contiguous multicarrier waveforms in practical opportunistic wireless systems. IET Radar, Sonar & Navigation.

[60] Prasad, R. (2014). *5G: 2020 and beyond*. River Publishers: Aalborg, Denmark.

[61] Rui, Y., Hu, H., Li, M., Zhang, X., Yi, H., & Yang, Y. (2009). Comparing effects of carrier frequency offset on generalized multi-carrier and OFDM systems. In *Proceedings of the IEEE international conference on communications (ICC), Dresden, Germany* (pp. 1–6).

[62] Sahin, A., Guvenc, I., & Arslan, H. (2013). A survey on prototype filter design for filter bank based multicarrier communications. http://arxiv.org/pdf/1212.3374.pdf.

[63] Schafhuber, D., Matz, G., & Hlawatsch, F. (2002). Pulse-shaping OFDM/BFDM systems for time-varying channels: ISI/ICI analysis, optimal pulse design, and efficient implementation. In *Proceedings of the 13th IEEE international symposium personal, indoor mobile radio communications (PIMRC), Lisbon, Portugal* (Vol. 3, pp. 1012–1016).

[64] Siohan P, Siclet C, Lacaille N (2002). Analysis and design of OFDM/OQAM systems based on filterbank theory. IEEE Transactions on Signal Processing.

[65] Stuber GL, Almalfouh SM, Sale D (2009). Interference analysis of TV-band whitespace. Proceedings of the IEEE.

[66] Sun S, Ju Y, Yamao Y (2013). Overlay cognitive radio OFDM system for 4G cellular networks. IEEE Wireless Communications.

[67] Sutton, P., Ozgul, B., Macaluso, I., & Doyle, L. (2010). OFDM pulse-shaped waveforms for dynamic spectrum access networks. In *Proceedings of the IEEE symposium on new Frontiers in dynamic spectrum (DySPAN), Singapore* (pp. 1–2).

[68] Tandra R, Mishra SM, Sahai A (2009). What is a spectrum hole and what does it take to recognize one?. Proceedings of the IEEE.

[69] Wang W, Yu G, Huang A (2013). Cognitive radio enhanced interference coordination for femtocell networks. IEEE Communications Magazine.

[70] Wannstrom, J. (2013). *Carrier aggregation explained*. http://www.3gpp.org/Carrier-Aggregation-explained.

[71] Weiss, T., Hillenbrand, J., Krohn, A., & Jondral, F. (2004). Mutual interference in OFDM-based spectrum pooling systems. In *Proceedings of the IEEE 59th vehicular technology conference (VTC 2004-Spring), Milan, Italy* (pp. 1873–1877).

[72] Wyglinski, A. M. (2006). Effects of bit allocation on non-contiguous multicarrier-based cognitive radio transceivers. In *Proceedings of the IEEE 64th vehicular technology conference (VTC 2006-Fall), Montreal, QC*.

[73] Xiao J, Hu RQ, Qian Y, Gong L, Wang B (2013). Expanding LTE network spectrum with cognitive radios: From concept to implementation. IEEE Wireless Communincations.

[74] Yap, K. K., Sherwood, R., Kobayashi, M., Huang, T. Y., Chan, M., Handigol, N., McKeown, N., & Parulkar, G. (2010). Blueprint for introducing innovation into wireless mobile networks. In *Proceedings of the 2nd ACM SIGCOMM workshop on virtualized infrastructure systems and architectures (VISA), New Delhi, India* (Vol. 3, pp. 25–32).

[75] Yucek T, Arslan H (2009). A survey of spectrum sensing algorithms for cognitive radio applications. IEEE Communincations on Surveys and Tutorials.

[76] Zakaria R, Ruyet DL (2012). A novel filter-bank multicarrier scheme to mitigate the intrinsic interference: Application to MIMO systems. IEEE Transactions on Wireless Communications.

[77] Zhang X, Zhou X (2012). LTE-advanced air interface technology.

